# Increased dosage and treatment time of Epigallocatechin-3-gallate (EGCG) negatively affects skeletal parameters in normal mice and Down syndrome mouse models

**DOI:** 10.1371/journal.pone.0264254

**Published:** 2022-02-23

**Authors:** Raza Jamal, Jonathan LaCombe, Roshni Patel, Matthew Blackwell, Jared R. Thomas, Kourtney Sloan, Joseph M. Wallace, Randall J. Roper

**Affiliations:** 1 Department of Biology, Indiana University-Purdue University Indianapolis, Indianapolis, Indiana, United States of America; 2 Department of Biomedical Engineering, Indiana University-Purdue University Indianapolis, Indianapolis, Indiana, United States of America; Nathan S Kline Institute, UNITED STATES

## Abstract

Bone abnormalities affect all individuals with Down syndrome (DS) and are linked to abnormal expression of *DYRK1A*, a gene found in three copies in people with DS and Ts65Dn DS model mice. Previous work in Ts65Dn male mice demonstrated that both genetic normalization of *Dyrk1a* and treatment with ~9 mg/kg/day Epigallocatechin-3-gallate (EGCG), the main polyphenol found in green tea and putative DYRK1A inhibitor, improved some skeletal deficits. Because EGCG treatment improved mostly trabecular skeletal deficits, we hypothesized that increasing EGCG treatment dosage and length of administration would positively affect both trabecular and cortical bone in Ts65Dn mice. Treatment of individuals with DS with green tea extract (GTE) containing EGCG also showed some weight loss in individuals with DS, and we hypothesized that weights would be affected in Ts65Dn mice after EGCG treatment. Treatment with ~20 mg/kg/day EGCG for seven weeks showed no improvements in male Ts65Dn trabecular bone and only limited improvements in cortical measures. Comparing skeletal analyses after ~20mg/kg/day EGCG treatment with previously published treatments with ~9, 50, and 200 mg/kg/day EGCG showed that increased dosage and treatment time increased cortical structural deficits leading to weaker appendicular bones in male mice. Weight was not affected by treatment in mice, except for those given a high dose of EGCG by oral gavage. These data indicate that high doses of EGCG, similar to those reported in some treatment studies of DS and other disorders, may impair long bone structure and strength. Skeletal phenotypes should be monitored when high doses of EGCG are administered therapeutically.

## Introduction

Trisomy 21 (Ts21) causes skeletal deficits in all individuals with Down syndrome (DS) that begin early in development, manifest in adolescence, and progress to osteoporosis. Individuals with DS present with a short stature, low bone mineral density (BMD), and increased fracture risk. Ts21 causes altered skeletal growth perinatally, a shorter period of bone growth that leads to reduced stature, earlier attainment of peak bone mass, and age-related bone loss sooner than typically developing individuals [1–4]. The skeletal deficits seen at younger ages in people with DS place these individuals at significant risk for bone fracture and osteoporosis at both younger and older ages [2,5–7]. Males as compared to females with DS seem to be more prone to earlier age of onset of skeletal deficiencies [8,9] Because the lifespan of people with DS has increased from an average of 20 years a few decades ago to 60 or more years presently [10,11], the burden of osteoporosis and fragility fractures has likewise increased.

The Ts65Dn mouse model, trisomic for about half of the gene orthologs found on human chromosome 21 (Hsa21) [12], recapitulates many of the phenotypes associated with DS [13,14]. These phenotypes include several skeletal phenotypes found in humans with DS including craniofacial deficits, small stature, reduced BMD, and trabecular (the three-dimensional latticework of bone present in the ends of long bones and in vertebrae and the iliac crest) and cortical (the solid shaft of long bones that gives strength) skeletal deficits [13,15–18]. Trisomic male mice show structural and mechanical skeletal deficits by 6 weeks of age. At all ages studied (6, 12, 16, and 104 weeks), percent bone volume (BV/TV) and trabecular number (Tb.N) were significantly lower, and trabecular separation (Tb.Sp) higher in male Ts65Dn compared to euploid control mice [13,15,19]. Cortical measures (e.g., cross-sectional area (CSA), cross-sectional perimeter, and mean polar moment of inertia) in femurs of 6- and 16-week-old male Ts65Dn mice were also significantly decreased [13,19]. Mechanical data in 6-week-old trisomic as compared to euploid mice showed significant decreases in ultimate force, stiffness, energy to failure, and toughness. Osteoblast (bone forming cell) activity and bone formation is lower in male Ts65Dn mice at 6 and 12 weeks. Osteoclast (bone resorbing cell) number is increased in 6-week-old but decreased in 12-week-old Ts65Dn as compared to euploid male mice [15,19].

Many phenotypes caused by Ts21, including deficiencies observed in bone, have been linked to the trisomic gene *Dual Specificity Tyrosine Phosphorylation-Regulated Kinase 1A* (*DYRK1A*) [19–21]. DYRK1A is a serine-threonine kinase that regulates many downstream proteins and transcription factors [20,22–24], and is found in three copies in many DS mouse models, including Ts65Dn. *Dyrk1a* transgenic mice (increased copy number of *Dyrk1a* alone) exhibited osteoporotic phenotypes including lower BV/TV, Tb.N, trabecular thickness, (Tb.Th) and increased Tb.Sp [25]. Increased *Dyrk1a* expression in vivo reduced osteoblast generation and decreased osteoclastogenesis but did not seem to affect the cortical bone thickness in *Dyrk1a* transgenic mice. Cortical bone formation rate (BFR) and mineral apposition rate (MAR) were lower in *Dyrk1a* transgenic mice. Returning *Dyrk1a* to two functional copies in otherwise trisomic 6-week-old Ts65Dn mice (Ts65Dn,*Dyrk1a*+/-) normalized BV/TV, Tb.N, Tb.Th, Tb.Sp, CSA, and trabecular and cortical BFR and MAR to euploid levels [19]. Removing one functional copy of *Dyrk1a* from osteoblasts of otherwise trisomic Ts65Dn mice did not improve trabecular or cortical bone phenotypes [26]. Trisomic *Dyrk1a* is linked to both DS-associated trabecular and cortical bone abnormalities and many other DS abnormalities and is a strong candidate for therapeutic targeting [21,27,28]. *Dyrk1a*, however, may not be the only trisomic gene responsible for all skeletal phenotypes associated with DS and other unidentified genes and signaling pathways likely contribute to the incidence and severity of DS associated bone abnormalities.

Epigallocatechin gallate (EGCG), one of many polyphenols found in green tea, inhibits DYRK1A in vitro [29], and has been proposed as a possible therapeutic for DS phenotypes. In addition to affecting DYRK1A, EGCG may also increase free radical scavenging activity and reduce oxidative stress, enhance bone mineralization, repress bone resorption, and improve both trabecular and cortical bone [30]. EGCG may affect body mass in animal models and humans [30], and treatment with green tea extracts (GTE) containing EGCG for 12 months been shown to improve body composition and cause weight loss in males with DS [31]. We previously showed that ~9 mg/kg/day EGCG given in water ad libitum for 3 weeks during adolescence (postnatal days (PD) 24–45) rescued trabecular bone phenotypes seen in Ts65Dn mice to euploid levels [19]. EGCG-treated trisomic male mice had increased cortical and trabecular MAR and trabecular BFR as compared to non-treated trisomic mice. Results from these EGCG-treated Ts65Dn male mice suggested increased osteoblast number and activity and decreased osteoclast number in trabecular bone. There were no significant effects of EGCG treatment on euploid littermate mice. Based on these results, we hypothesized that a higher dosage or extended treatment of EGCG in Ts65Dn mice would further improve bone deficits, especially in cortical bone, associated with DS. Because early bone deficits are more pronounced in males with DS and have been documented in Ts65Dn male mice, and weight loss had been observed in males with DS after GTE treatment, we analyzed treatment of Ts65Dn and control male mice with ~20 mg/kg/day EGCG for 7 weeks and compared these results with previously published results from ~9, 50, and 200 mg/day EGCG treatment of male Ts65Dn and control mice.

## Materials and methods

### Animals

Female B6EiC3Sn a/A-Ts(17^16^)65Dn (Ts65Dn—Stock #001924) and male B6C3F1 (Stock #100010) mice were purchased from the Jackson Laboratory (Bar Harbor, ME, USA).(Ts65Dn × B6C3F1) matings were continuously bred in our colony in rooms with a standard 12:12 light:dark cycle to generate and maintain the mice used in the study. New Ts65Dn and B6C3F1 mice from the Jackson Laboratory were added to the breeding colony every 6–12 months during all the experiments. Ts65Dn male mice are sterile, and because of the need to use female mice to maintain the breeding colony, only male mice were used in these studies. Additionally, previous DS associated bone deficits have been characterized in 6-week-old male Ts65Dn mice and sample size in each group was dictated by these previous experiments [8]. Mice were genotyped by PCR according to established protocols [32]. Male mice in the 20mg/kg/day EGCG study were randomly selected from 27 litters with an average size of 4.9 total and 2.8 male mice (at postnatal day 10) per litter. Mice from these same litters were used for other studies and for colony propagation. Mice were weaned between postnatal days 21 and 24 and males were housed separately until beginning treatment at P24. The study of 20 mg/kg/day EGCG treatment utilized 55 male animals; 36 mice in the skeletal study and 45 mice in the weight study. Broken bones and weights that were not recorded led to not including animals in both studies. For health and welfare of the mice, general procedure in the colony is that if animals become sick or distressed (decreased mobility, decreased appetite, reluctance to move, a hunched or ungroomed appearance, problems with birth including dystocia, and displaying any type of wound), a veterinarian consultation is requested, or the animal is euthanized at that point and removed from the study. For this study, no animals were euthanized because they became sick or distressed for the duration of the experiments. Experiments with animals were carried out in accordance with the NIH Guide for the Care and Use of Laboratory Animals and received prior approval from the IUPUI School of Science Institutional Animal Care & Use Committee (IACUC) (SC213R and SC255R).

### Treatment

Treatment with ~20 mg/kg/day EGCG (>95% purity, as determined by LC/MS analyses [33]) was prepared by making a stock solution of 15 mg/mL EGCG in phosphate buffered saline (PBS). Treatments were delivered via drinking water in a concentration of 0.124 mg/mL EGCG, prepared by diluting the stock solution in tap water, and adding H_3_PO_4_, to a pH of ~5.5 to stabilize the EGCG in solution [34,35]. Treatments started on PD 24, usually 3 days after weaning. Treatments (EGCG or acidified water control) were randomized for the animals and were placed in drinking tubes and the mice (one mouse per cage with a 5cm^2^ cotton fiber nestlet) were allowed access ad libitum to its designated treatment as its sole source of fluid. The treatments were changed every 48 hours and at this time volumes consumed, and animal weights were recorded. Treatments continued throughout the duration of behavioral testing, which ended on PD 68.

### Bone extraction and analysis

Mice treated with 20 mg/kg/day EGCG or vehicle (randomly assigned) were euthanized at PD 68 by inhalation of isoflurane followed by cervical dislocation. Left and right femurs were extracted either at the time of death or after the carcass was frozen at -20°C. Femurs were wrapped in gauze soaked in PBS and stored in a -20°C freezer, or ethanol and stored at room temperature. Bone analysis for mice given 20 mg/kg/day EGCG or vehicle was performed as described in [36]. Briefly, femurs were scanned using a high-resolution micro computed tomography (μCT) system (SkyScan 1172, Bruker microCT, Belgium) that was calibrated using two cylindrical hydroxyapatite phantoms (0.25 and 0.75 g/cm^3^ CaHA) prior to each scanning session. Femurs from the 20 mg/kg/day EGCG treatment group were thawed then scanned from the distal condyle to the third trochanter using 60kV, 12μm resolution, and Al 0.5mm filter. Scans were reconstructed and rotated using NRecon and Dataviewer (SkyScan, Bruker microCT, Belgium), respectively. Reconstructed bones were then analyzed using CT analyzer (SkyScan, Bruker microCT, Belgium) and MatLab (MathWorks, Inc. Natick, MA) to obtain parameters for bone mineral density (BMD), percent bone volume (BV/TV), trabecular thickness (Tb.Th), trabecular separation (Tb.Sp), trabecular number (Tb.N), total cortical cross-sectional area (Tt.Ar.), cortical area (Ct.Ar.), cortical thickness (Ct.Th), periosteal bone surface (Ps.BS), endocortical bone surface (Es.BS), and marrow area (Ma.Ar). The trabecular region of interest (ROI) was calculated based on the overall size of the bone and a distance of 10% of the total bone length beginning at the proximal end of the distal growth plate and extending distally and were obtained through a custom Matlab code that excludes the outer cortical bone. Measurements of trabecular microarchitecture were calculated via CTan. The cortical ROI was calculated as a region of seven transverse slices at 60% of the bone’s overall length away from the proximal end of the growth plate. Using Matlab and a custom code, the cortical ROI was used to measure geometric properties [36,37].

### Statistical analysis

The skeletal measurements had normal or near-normal distributions. The bone parameters were analyzed with two-way ANOVAs using genotype and treatment as between-subjects factors. For each parameter with a significant interaction, post hoc analyses (Tukey’s multiple comparisons test) determined differences between groups.

In the combined analysis for each treatment dosage, treated trisomic and euploid mice were normalized to their control counterparts. Results of skeletal analysis shown in the tables were normalized as mice receiving EGCG divided by control mice. Due to euthanizing mice at different ages in the combined studies, weight data were normalized to kg/week of age.

### Comparison samples

Comparison details of ~9, 50, or 200 mg/kg/day (published) or 20 mg/kg/day (this article) EGCG treatments of Ts65Dn and control mice are given in Table 1 [19,35,36,38]. EGCG treatment methodology was as described in Table 1. H_3_PO_4_ was added 20 and 50 mg/kg/day EGCG treatment to stabilize the EGCG in solution [34,35]. Although the method used to scan the 20 mg/kg/day EGCG-treated femurs was the same as the 200 mg/kg/day study [38], this method differed slightly for the 50 mg/kg/day EGCG study [36]. These bones were scanned using a 6 μm resolution as well as using binning mode 2k. The 9 mg/kg/day EGCG study used dual energy X-ray absorptiometry (DXA) and μCT to obtain BMD. Cortical and trabecular analyses for the 9 mg/kg/day femurs were conducted using a different analysis and parameters of interest in cortical bone were not calculated aside from total cortical surface area [19].

**Table 1:** EGCG treatment, duration and methodology.

	Euploid + PBS (N)	Euploid + EGCG (N)	Ts65Dn + PBS (N)	Ts65Dn + EGCG (N)	Treatment Start Age	Treatment Duration	Treatment Method	Treatment stabilized with H_3_PO_4_	Age at euthanasia
**9 mg/kg/day** ^**a**^	11	10	10	10	3 Weeks	3 Weeks	Ad libitum	-	42 days
**20 mg/kg/day** ^**b**^	12	7	10	7	3 Weeks	7 Weeks	Ad libitum	+	68 days
**50 mg/kg/day** ^**c**^	14	9	9	11	3 Weeks	7 Weeks	Ad libitum	+	68 days
**200 mg/kg/day** ^**d**^	13	12	6	9	6 Weeks	3 Weeks	Oral gavage	-	68 days

Data from

^a^ Blazek et al. 2015

^b^ This study

^c^ Stringer et al. 2017, and

^d^ Goodlett et al 2020.

## Results

### Treatment with 20 mg/kg EGCG for 7 weeks

**Fig 1:**
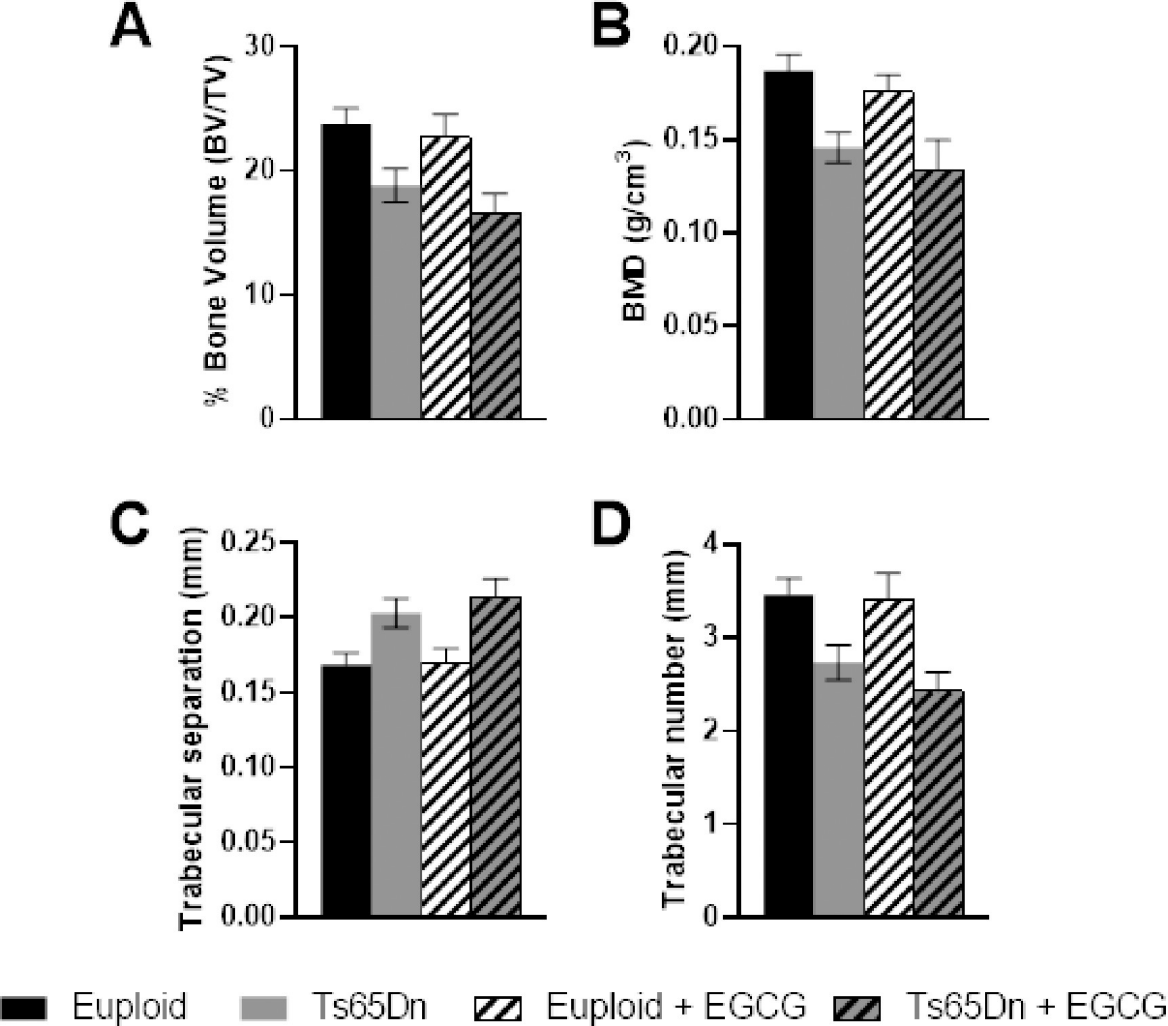
Trabecular bone measurements from Ts65Dn and Euploid mice treated with EGCG or control fluid. A. Percent bone volume. B. Bone mineral density (BMD). C. Trabecular separation (Tb.Sp). D. Trabecular number (Tb.N). Numbers indicated are average measurements ± standard error of the mean (SEM). No differences were detected between groups in post hoc analyses. For numbers of animals and ANOVA results, please see Table 2.

**Fig 2:**
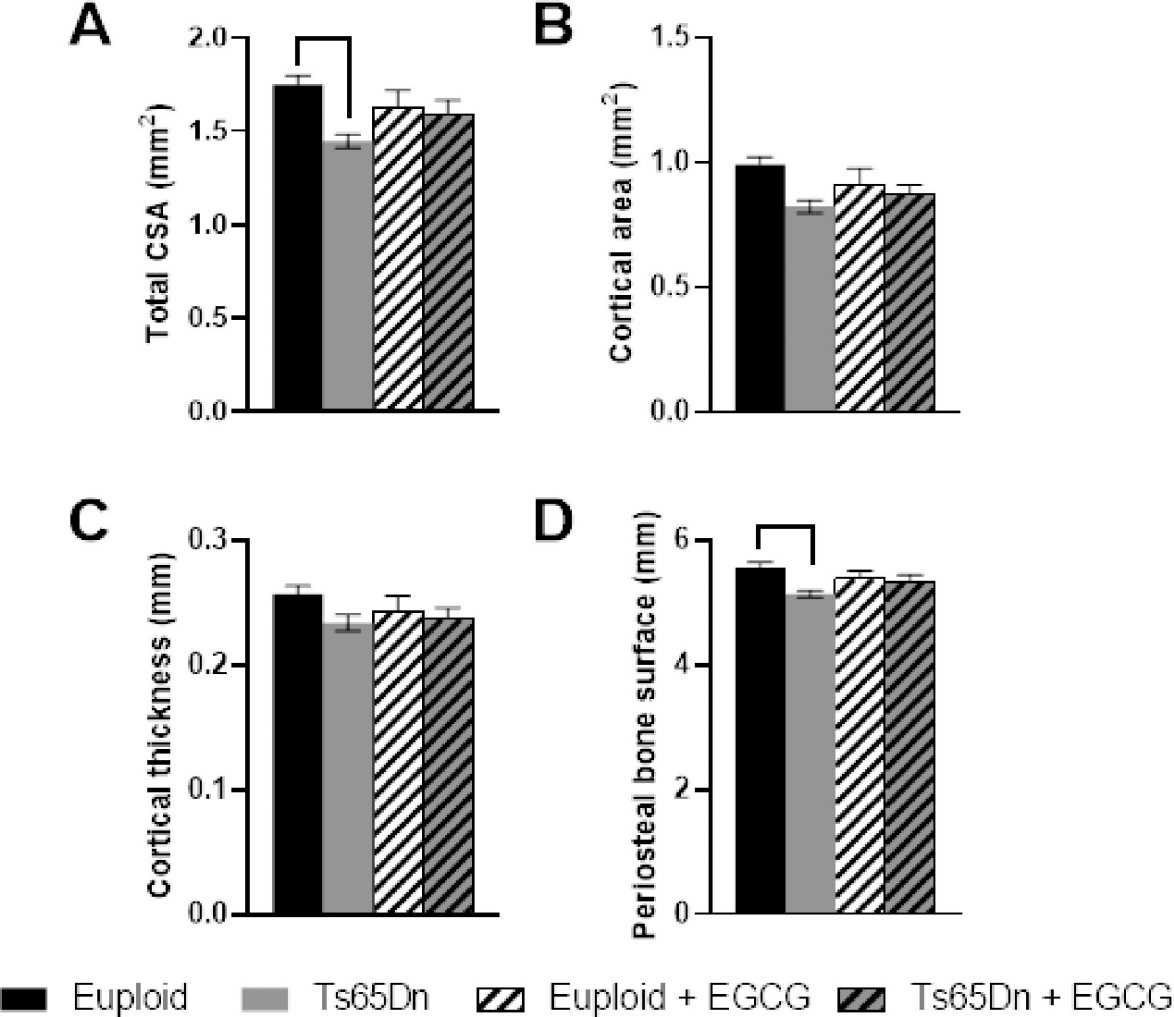
Cortical bone measurements from Ts65Dn and Euploid mice treated with EGCG or control fluid. A. Total cortical surface area (CSA). B. Cortical area (Ct.Ar). C. Cortical Thickness (Ct.Th). D. Periosteal bone surface (Ps.BS). Numbers indicated are average measurements ± standard error of the mean (SEM). Bars indicated differences between groups in post hoc analyses. For numbers of animals and ANOVA results, please see Table 2.

In Ts65Dn and littermate control male mice treated with ~20 mg/kg/day EGCG or vehicle, beginning at 3 weeks of age and lasting 7 weeks, trisomic mice (treated and untreated combined) displayed a genotypic effect with negative results in trabecular architecture compared with euploid mice (treated and untreated combined) (Table 2 and Fig 1). Control mice had higher BMD, BV/TV, and Tb.N. Trabecular separation (Tb.Sp) increased in trisomic as compared to euploid mice (Fig 1A–1D). Like other comparisons between Ts65Dn and euploid mice, trabecular thickness (Tb.Th, Table 2) was not significantly different. There were no significant effects of 20 mg/kg/day EGCG treatment on trabecular bone. In cortical bone, there were significant interactions between genotype and treatment for total CSA and periosteal bone surface (Ps.BS) (Table 2 and Fig 2). In post-hoc analyses, euploid control bones exhibited a higher total CSA and Ps.BS than trisomic untreated bones (Fig 2). No other cortical parameters showed differences in genotype or treatment effects between samples.

**Table 2:** Parameters of Skeletal Structure for Ts65Dn and Euploid mice treated with 20 mg/kg/day EGCG or vehicle (Average ± SEM; ANOVA analysis).

Skeletal structure	Eu+H_2_O	Eu+EGCG	Ts+H_2_O	Ts+EGCG	P value	P value	P value
	(n = 12)	(n = 7)	(n = 10)	(n = 7)	Genotype	Treatment	Interaction
**Trabecular bone**							
Percent bone volume (BV/TV) (%) ^a^	23.782 ± 1.247	22.730 ± 1.821	18.839 ± 1.332	16.608 ± 1.552	**0.0008**	0.2786	0.6950
Bone mineral density (BMD) (g/cm^3^) ^a^	0.187 ± 0.009	0.1776 ± 0.009	0.146 ± 0.008	0.134 ± 0.016	**0.0005**	0.2881	0.9693
Trabecular thickness (Tb.Th) (μ)	0.069 ± 0.001	0.067 ± 0.001	0.069 ± 0.002	0.068 ± 0.002	0.0532	0.3905	0.6743
Trabecular separation (Tb.Sp) (mm) ^a^	0.168 ± 0.008	0.170 ± 0.009	0.203 ± 0.010	0.214 ± 0.012	**0.0004**	0.5188	0.6543
Trabecular number (Tb.N) (1/mm) ^a^	3.463 ± 0.170	3.416 ± 0.271	2.732 ± 0.188	2.427 ± 0.202	**0.0002**	0.4028	0.5397
**Cortical Bone**							
Total cross-sectional area (CSA) (Tt.Ar) (mm^2^) ^a^^,^^c^	1.750 ± 0.050	1.628 ± 0.091	1.447 ± 0.036	1.589 ± 0.078	**0.0095**	0.8667	**0.0407**
Marrow area (Ma.Ar) (mm^2^)	0.757 ± 0.035	0.714 ± 0.051	0.624 ± 0.023	0.712 ± 0.059	0.1078	0.5828	0.1148
Cortical area (Ct.Ar) (mm^2^) ^a^	0.993 ± 0.029	0.914 ± 0.061	0.823 ± 0.026	0.877 ± 0.034	0.091	0.7464	0.0857
Cortical thickness (Ct.Th) (mm)	0.257 ± 0.006	0.243 ± 0.013	0.234 ± 0.007	0.237 ± 0.009	0.0908	0.5277	0.3223
Periosteal BS (Ps.BS) (mm) ^a^^,^^c^	5.564 ± 0.080	5.385 ± 0.128	5.134 ± 0.054	5.339 ± 0.019	**0.0129**	0.9150	**0.0468**
Endocortical BS (Es.BS) (mm)	3.930 ± 0.137	3.755 ± 0.117	3.552 ± 0.058	3.725 ± 0.138	0.1088	0.9930	0.1687
Tissue mineral density (TMD) (g/cm^3^ HA)	1.078 ± 0.007	1.071 ± 0.010	1.087 ± 0.011	1.075 ± 0.012	0.5137	0.3587	0.8331

^a^ Main effect of genotype

^b^ main effect of treatment (EGCG vs. water)

^c^ Interaction of genotype × treatment.

### Effects of increasing dosage and time of EGCG treatment

Because of the minimal effects observed in cortical bone with ~9mg/kg/day EGCG [19] and some positive effects of ~20mg/kg/day EGCG in trisomic mice, we reasoned that a longer and higher dose of EGCG treatment may improve cortical bone in trisomic animals. Increasing the dose of EGCG to 50 mg/kg/day for 7 weeks or 200 mg/kg/day for 3 weeks (as previously published) [36,38], however, did not improve the total CSA or Ps.BS in trisomic mice and significantly reduced Ct.Ar (p<0.05, treatment effect) and Ct.Th (p<0.05) in trisomic mice, in both studies. As previously published, mice that received 200 mg/kg/day also had increased marrow area (Ma.Ar) and endocortical bone surface (Es.BS). Because cortical measurements generally decreased with increasing EGCG dosage, we hypothesized that there was a negative effect of increasing EGCG dose and duration of treatment on cortical bone. To test this hypothesis, a percent change was calculated to compare the effects of EGCG treatment between studies. Cortical parameters in ~9, 20, 50 and 200 mg/kg/day EGCG studies were compared by normalizing the treated skeletal values by the untreated values in both euploid and trisomic mice (Table 3). Less emphasis was placed on results from the ~9 mg/kg/day EGCG treatment study because of the low EGCG dosage, short treatment time, age of mice at death, and different skeletal analysis methodologies. In both euploid and trisomic mice, Ct.Th decreased in treated trisomic and euploid male mice (as a function of control treated mice) as the EGCG dosage increased (Table 3). Tt.Ar, Ma.Ar, Ct.Ar, Ps.BS, and Es.BS slightly increased in treated euploid male mice and slightly decreased in treated trisomic male mice (as a function of mice that received no treatment). TMD, however, was largely unchanged in treatments of ~20, 50, and 200 mg EGCG. In summary, 9 mg/kg/day EGCG had minimal effect on trisomic cortical bone, 20 mg/kg/day EGCG showed some positive effects in trisomic cortical bone, but 50 mg/kg/day EGCG treatment showed no change or slightly reduced effects in trisomic and euploid cortical bone. Treatment with 200 mg/kg/day EGCG showed some positive effects in euploid male mice, but mostly negative effects on cortical bone in trisomic male mice (Table 3).

**Table 3:** Normalized (treated/untreated) skeletal measurements used to compare values between studies with different treatment methodologies.

	9 mg/kg/day ^a^	20 mg/kg/day	50 mg/kg/day	200 mg/kg/day
**Total cross-sectional area (CSA) (Tt.Ar)**				
**Euploid**	1.06	0.93	0.96	1.02
**Trisomic**	0.98	1.10	0.97	0.93
**Marrow Area (Ma.Ar)**				
**Euploid**	ND	0.94	1.01	1.13
**Trisomic**	ND	1.14	1.00	1.00
**Cortical Area (Ct.Ar)**				
**Euploid**	1.05	0.92	0.92	0.95
**Trisomic**	1.06	1.07	0.96	0.88
**Cortical Thickness (Ct.Th)**				
**Euploid**	ND	0.95	0.93	0.91
**Trisomic**	ND	1.01	0.96	0.89
**Periosteal Bone Surface (Ps.BS)**				
**Euploid**	0.98	0.97	0.97	1.01
**Trisomic**	0.99	1.04	1.00	0.97
**Endocortical Bone Surface (Es.BS)**				
**Euploid**	ND	0.96	1.00	1.06
**Trisomic**	ND	1.05	1.00	0.99
**Tissue Mineral Density (TMD)**				
**Euploid**	ND	0.99	1.00	0.99
**Trisomic**	ND	0.99	0.99	0.97

ND = measurement not done in the original study. 1.00 = trisomic measurement is equal to that observed in euploid treated animals.

^a^ Animals treated with ~9 mg/kg/day EGCG were only treated for 21 days and were euthanized at 42 days.

### Weight changes with EGCG treatment

**Fig 3:**
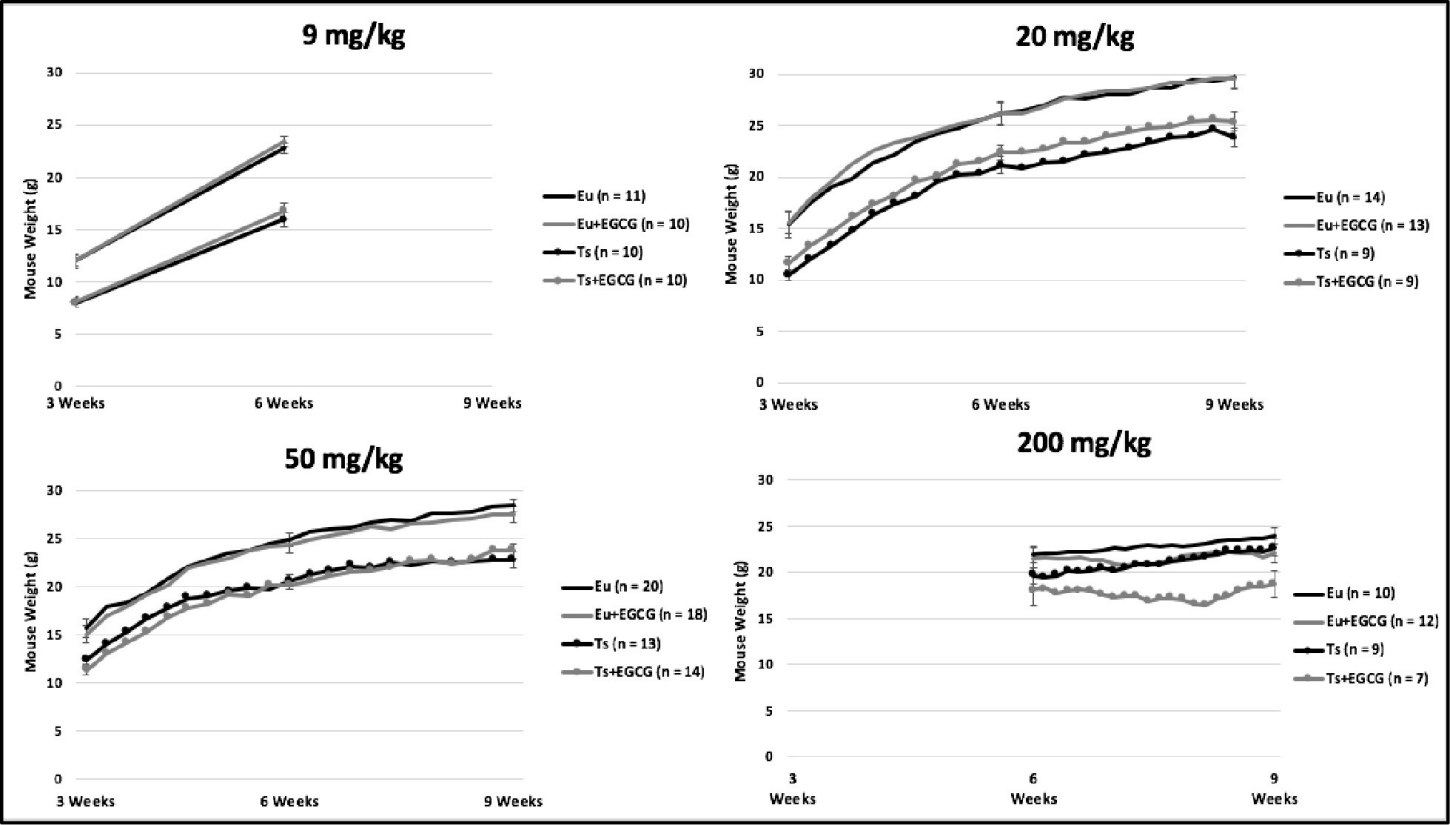
Weights of Ts65Dn (Ts) and Euploid (Eu) mice treated with EGCG or control fluid. Weights were taken at times indicated by the dots on the graphs of the Ts mice. Standard error of the mean (SEM) measurements given at 3, 6, and 9 weeks at times weights were measured.

Weight over treatment time was examined in trisomic and euploid mice with and without EGCG treatment. In experiments where ~9, 20, and 50 mg/kg EGCG was given ad libitum in the drinking water, weight generally increased over time in both euploid and Ts65Dn mice, though weight was lower in all Ts65Dn as compared to all euploid male mice (Fig 3). In male mice that were treated by gavage with 200 mg/kg/day EGCG or vehicle, neither trisomic nor euploid mice showed a significant increase in weight over time as did mice from the 20 or 50 mg/kg/day ad libitum EGCG treatments. Additionally, trisomic mice given 200 mg/kg/day EGCG through gavage had significantly less mass than trisomic mice given vehicle at 9 weeks of age.

## Discussion

### EGCG treatment for down syndrome related phenotypes

Individuals with DS are being given EGCG or GTE containing EGCG to alleviate cognitive deficits associated with DS [39–42]. Small clinical trials of 3–12 months reported some improvements in cognitive parameters including visual recognition memory after 3 months of treatment [40] and visual recognition immediate memory; inhibitory control and adaptive behavior after twelve months of treatment [39] with GTE containing ~7–13 mg/kg/day EGCG. In a survey of caregivers of individuals with DS, the caregivers reported giving doses of EGCG that ranged from 1–2000 mg/day (average of ~351 mg/day), at an average age of under 10 years, with some receiving EGCG as newborns. For those who provided weight of individuals with DS receiving green tea extracts or EGCG, the average dose of EGCG was between 10–30 mg/kg/day [42]. These numbers of individuals receiving EGCG or EGCG-containing GTE and the doses given warranted examination of different doses of EGCG on other parameters it has been shown to affect.

### Effects on bone of different doses of EGCG

Commercially available green tea and GTE that individuals with DS may use or found in studies with DS mouse models contain EGCG concentrations that vary from product to product. Treatment with GTE containing EGCG has showed improvements in some neuroanatomical and neurobehavioral measurements in DS mouse models [43–47]. For bone, in mouse models of DS given GTE with different concentrations of EGCG, there was limited improvement in BV/TV, and some other trabecular parameters, but overall bone strength was decreased [33]. When ~9 mg/kg/day pure EGCG was administered from 3–6 weeks of age in male mice, there were improvements in trabecular bone and increased bone toughness, but other mechanical and material properties were not enhanced. Trabecular parameters were not improved in Ts65Dn male mice given ~20 mg/mg/day. Increasing the dosage of EGCG to 20 mg/kg/day and length of treatment to 7 weeks showed an interactive effect with some improvements in total CSA and Ps.BS in trisomic mice). When multiple treatments of increasing dosages of EGCG were examined together, the overall cortical thickness in trisomic and euploid male mice was reduced as EGCG dosage increased. Higher doses of EGCG treatment resulted in a differential effect on trisomic and euploid mice, with trisomic cortical parameters generally reduced and euploid parameters as EGCG treatment increased in male mice. TMD was largely unchanged with increased EGCG dosage. The reduction of cortical bone parameters in trisomic male mice may lead to reduced bone strength. Based on the results of these multiple analyses of the effects of EGCG on bone in the Ts65Dn DS mouse model, these findings suggest that lower doses of EGCG for shorter treatment durations may improve trabecular parameters in long bones including the femur but may not lead to improved overall bone strength. Higher doses of EGCG may negatively affect bones, especially with trisomic material.

### Effects on weight of different doses of EGCG

Although weights of male Ts65Dn mice were lower than their euploid counterparts, and they increased over time, similar to prior measurements [48], the weights of trisomic or euploid male mice treated with ~9, 20 or 50 mg/kg/day EGCG were not significantly affected. Male mice administered 200 mg/kg/day EGCG via oral gavage, however, did not show increases in weight over time, and all trisomic and euploid mice that received 200 mg/kg/day EGCG had significantly lower mass after 3 weeks of treatment. From these data, we conclude that the stress of the gavage procedure most likely kept all mice receiving 200 mg/kg/day EGCG from significant weight gain during treatment, and there is a significant effect of EGCG treatment that subdued weight gain as the 200 mg/kg/day EGCG treatment continued.

### Putative effects of green tea and/or EGCG on bone and weight in humans

Green tea has been hypothesized to contribute to beneficial effects in bone health and a positive correlation between tea drinking and increased BMD has been reported in multiple studies of postmenopausal women [49–55]. No adverse side effects have been reported in habitual green tea drinkers, and EGCG crosses both placental and blood-brain barriers, exhibits low toxicity, and absorbs in the gastrointestinal tract [44,56,57]. Green tea polyphenols given to female, female ovariectomized, and aged male orchiectomized rats generally increased BMD, BV/TV, trabecular microarchitecture, BFR and MAR [58,59]. These results contrast those reported from individuals with DS or DS mouse models given GTE or EGCG. When individuals with DS were given GTE containing EGCG (calculated to ~7-13mg/kg/day), the bone mass in females with DS was reduced after 12 months but recovered after six months after treatment cessation [31].

Several association studies have shown that consumption of tea is correlated with improved BMD, but fewer studies have shown that tea consumption reduces risk of bone fracture. These association studies often lack specific types and quantities of tea consumed, utilize a wide age window of participants, do not address mechanisms of action, and are usually done in postmenopausal women, because these individuals are more likely to have bone loss [60,61]. One study showed that green tea consumption was one of the factors linked to significantly higher BMD in women ≥60 years who attended an osteoporotic outpatient clinic [51]. Another case-control study associated green tea consumption as one of the factors that reduced hip fracture in 65–89 year old individuals adjusted for sex, age and BMI [62]. In a study of ~450,000 individuals aged 30–79 years who were hospitalized for a bone fracture, both men and women who self-reported drinking green tea daily and those that drank any type of tea for greater than 30 years had reduced risk of hip fracture [63]. In a subset of this cohort those who mostly drank green tea, there was an association with prolonged weekly consumers of tea and increased calcaneus BMD in women but not men [64]. The beneficial effects on bone were not seen in women who drank more than five cups (1 cup = 300 ml) of tea per day, and no detrimental effects on bone were seen in any of the tea drinkers. Other studies of habitual tea drinkers, the majority of which drank green tea, and were 20 years of age or older, show improved BMD [52].

Following these correlative studies and numerous animal studies that showed that green tea improved bone properties, prospective studies in humans have shown that green tea may improve some bone properties. Most prospective studies in humans and mice examining skeletal properties utilize GTE and not pure EGCG and administer EGCG doses far lower than those reported in this study [30]. Postmenopausal osteopenic women given either 500 mg/day (4–6 cups of green tea polyphenols (GTP)), as compared to placebo, for six months showed improved bone specific alkaline phosphatase (BAP) levels (an indicator of bone formation) at one month in those that received GTP, and a higher ratio of BAP to tartrate-resistance acid phosphatase (TRAP—bone degradation marker) at three and six months in those that received GTP and engaged in Tai Chi exercise [65]. Postmenopausal women that were overweight and obese were given GTE containing 843 mg EGCG (approximately five 240 ml servings of brewed green tea) without caffeine or control treatment for 12 months. This intervention did not affect overall adiposity, fat-free mass, or BMD in women assessed in this study [66]. Our work suggests that some positive effects seen in normal individuals given EGCG may not be observed, or may cause some harm, in trisomic individuals, especially as EGCG doses are increased.

In the study of individuals with DS receiving GTE with ~7–13 mg/kg/day EGCG for 12 months, males exhibited some weight loss [31]. Several studies have associated GTE treatment with weight loss in humans in general, but these studies were mostly done in obese women. GTE treatment (857 mg EGCG, decaffeinated) or control capsules for 12 weeks in obese women (average age 44 years) resulted in decreases in weight, BMI, waist circumference, cholesterol, LDL levels as compared with baseline levels [67]. When compared to a control group of women receiving placebo, there were no significant differences in body weight, BMI or waist circumference after treatment, but there were lower ghrelin and cholesterol and higher adiponectin levels detected in the group receiving EGCG as compared to placebo. A previous study of GTE treatment (302 mg EGCG and ~9 mg caffeine) or placebo for 12 weeks in obese women (16–60, average age 43 years) showed those receiving GTE exhibiting reduction in waist circumference and LDL cholesterol and increased in HDL cholesterol, adiponectin, and ghrelin when compared to baseline levels, but no significant differences in weight, BMI, and waist circumference and no significant changes in obesity-related hormone levels when compared with a control group [68].

GTE treatment with significant caffeine levels may increase weight loss in individuals [69]. GTE treatment (~140 mg EGCG and 111 mg caffeine) for 24 weeks showed improved weight reduction in a small sample of less physically active older adults (average age = 69 years) [70]. A 12-week study of men and women (average age 49 years) found more weight loss in individuals receiving GTE (34 mg EGCG and 29 mg caffeine) as compared to control [71]. The caffeine in GTE may enhance the sympathetic nervous system, regulate appetite, or increase energy expenditure to cause the weight loss [72]. Depending on whether GTE treatment given to individuals with DS includes caffeine may affect weight differently in this selected population.

### Limitations of these data

There are several caveats that should be considered with these data. All these studies were done using male mice and there is a sexual dimorphism in the development of skeletal deficits between males and females with DS and in DS mouse models [8,9,26]. Although no weight changes were seen in females with DS given GTE, a loss in bone mass was observed [31]; GTE and EGCG treatment may affect bone and weight differently in females with DS and female DS mouse models. Mice receiving ~9 mg/kg/day EGCG were euthanized and had skeletal observations done at 6 weeks of age, whereas mice receiving 20, 50 or 200 mg/kg/day EGCG had skeletal parameters measured at 68 days (Table 1). Because the studies were done at different times, the parameters for measuring skeletal properties may have differed between studies, and we were not able to compare absolute values for each trait between studies. Additionally, we are not able to discount genetic drift in Ts65Dn mice over time as has been observed in neurodevelopmental studies in different Ts65Dn mouse models [73]. Moreover, to stabilize EGCG bioavailability [34], H_3_PO_4_ was added to EGCG in mice given 20 and 50 mg/kg EGCG per day; because EGCG was given by oral gavage in mice receiving 200 mg/kg/day, it was expected that the concentration received was close to the desired given concentration. These treatment differences likely altered the actual bioavailable EGCG received by mice [36,38]. Treatment given at earlier times may have had different effects on bone measurements including those mice receiving 9 mg/kg/day EGCG at 3 weeks of age; giving EGCG from prenatal and perinatal stages, respectively, improved craniofacial skeleton and some structural brain abnormalities [47,74]. Yet, EGCG given during older ages, likely would not affect craniofacial bone parameters as we have previously shown [19].

### Conclusions

Contrary to most published studies of the effects of GTE and EGCG on bone and weight that examine individuals at older ages, most individuals with DS that receive GTE or EGCG are of younger ages [39,40,42]. It is not known how high doses of EGCG affect the bones or weight of younger individuals. From the study giving GTE (decaffeinated) to individuals with DS, males with DS exhibited weight loss and females had a loss in bone mass [31]. In this small clinical trial on individuals with DS, it appears that an interaction between trisomy, the male sex and GTE caused the weight loss (in absence of caffeine). It is important to consider that the molecular mechanisms that regulate metabolism and bone mass may be affected by increased gene dosage; benefits observed in the general population may not have the same result in individuals with DS. Composition, gender, frequency, and duration of GTE/EGCG consumption may influence specific factors related skeletal homeostasis including geometry, bone mass, and risk of fractures. The information provided herein suggests that high doses of EGCG for long periods of time in developing male mice may reduce weight gain and/or bone mass and may portend similar effects in both weight and bone mass in individuals with DS. Taken together, these data suggest that the weight and bone mass of younger individuals with DS who receive GTE or EGCG should be monitored during and after administration of these nutraceuticals. These combined studies demonstrate limited benefits and known risks resulting from the interaction between trisomic genes and large doses of GTE or EGCG over long periods of time.

## Supporting information

S1 Data (XLSX)
